# Cumulative Adversity and Depressive Symptoms: Optimism as a Moderator

**DOI:** 10.1093/geroni/igaa057.1625

**Published:** 2020-12-16

**Authors:** Gabriella Dong, Mengting Li

**Affiliations:** Rutgers University, New Brunswick, New Jersey, United States

## Abstract

Older immigrants encountered a wide range of traumatic events across the life span, before and after immigration, in the origin and host countries. Prior studies have shown that traumatic events were associated with depression, but less is known about what are the resilience factors against depression related to traumatic events. Optimism is one of resilience assets reflecting the extent to which people hold generalized favorable expectancies for their future. This study aims to examine whether optimism could moderate the negative impact of exposure to natural disasters, traumatic personal events, and historical events on depressive symptoms. The data were drawn from the Population Study of Chinese Elderly in Chicago (PINE) in 2017-2019, with a sample size of 3,125. Traumatic life events were evaluated by natural disasters (typhoon, earthquake, and tornado), personal events (e.g. death of a loved one, physical assault) and historical events (e.g. Japanese invasion of China). Depressive symptoms were measured by PHQ-9. Optimism was assessed by Revised Life Orientation test. Linear regression with interaction terms was used. Older adults with one additional exposure to natural disaster (b=0.34, SE=0.07, p <.001), personal event (b=0.32, SE=0.05, p <.001), and historical event (b=0.14, SE=0.04, p <.001) were associated with higher depressive symptoms. Optimism could buffer the negative impacts of natural disasters (b=-0.03, SE=0.01, p <.05), personal events (b=-0.05, SE=0.01, p <.001), and historical events (b=-0.02, SE=0.01, p <.01) on depressive symptoms. Psychological interventions are suggested to increase optimism of older adults with exposure to lifetime traumatic events to reduce their depressive symptoms.

